# The Anti-Inflammatory Drug Aspirin Does Not Protect Against Chemotherapy-Induced Memory Impairment by Paclitaxel in Mice

**DOI:** 10.3389/fonc.2020.564965

**Published:** 2020-12-14

**Authors:** Aeson Chang, Ni-Chun Chung, Adam J. Lawther, Alexandra I. Ziegler, David M. Shackleford, Erica K. Sloan, Adam K. Walker

**Affiliations:** ^1^ Drug Discovery Biology Theme, Monash Institute of Pharmaceutical Sciences, Monash University, Parkville, VIC, Australia; ^2^ Laboratory of ImmunoPsychiatry, Neuroscience Research Australia, Randwick, NSW, Australia; ^3^ Centre for Drug Candidate Optimisation, Monash Institute of Pharmaceutical Sciences, Monash University, Parkville, VIC, Australia; ^4^ Division of Cancer Surgery, Peter MacCallum Cancer Centre, East Melbourne, VIC, Australia; ^5^ School of Psychiatry, University of New South Wales, Randwick, NSW, Australia

**Keywords:** cancer, cognitive impairment, memory, inflammation, anti-inflammatory drugs (NSAIDs)

## Abstract

Inflammation has been proposed to play a causal role in chemobrain which—if true—would represent an opportunity to repurpose existing anti-inflammatory drugs for the prevention and treatment of chemobrain. Here, we show that the chemoagent paclitaxel induces memory impairment and anhedonia in mice within 24 h of treatment cessation, but inflammation is not present until 2 weeks after treatment. We find no evidence of brain inflammation as measured by cytokine analysis at any time point. Furthermore, treating with aspirin to block inflammation did not affect paclitaxel-induced memory impairment. These findings suggest that inflammation may not be responsible for memory impairment induced by paclitaxel. These results contrast with recent findings of a causal role for inflammation in cancer-induced memory deficits in mice that were prevented by treatment with oral aspirin, suggesting that cognitive impairment in cancer patients undergoing treatment may arise from multiple convergent mechanisms.

## Introduction

Cognitive impairment and mood disturbance are widespread in cancer patients and survivors who have received chemotherapy. Colloquially called “chemobrain”, up to 70% of cancer patients report noticeable deficits in learning, memory, executive function and concentration after chemotherapy, and up to 40% have measurable cognitive decline (1–4). Roughly 20% of cancer patients experience mood-related disturbances including anxiety and depression (5). In many survivors these symptoms are sustained for months or years after cessation of treatment (6, 7). While the clinical phenomenon of chemotherapy-associated cognitive impairment and mood disorders has been widely characterized across a number of chemotherapies and different cancer populations, the biological mechanisms responsible for these symptoms remain elusive. A primary hypothesis proposed to account for sustained cognitive impairment and mood-related disorders is that chemotherapy drives peripheral inflammation that is propagated to the brain to induce neuroinflammation, thereby driving changes in cognition and mood. This “inflammation hypothesis” has been supported by findings of cognitive and mood symptoms in cancer patients and survivors that correlate with increases in proinflammatory cytokines and neutrophil-to-lymphocyte ratios during and after chemotherapy (6, 8). However, other studies have failed to find any association (3, 9). Such a hypothesis is plausible given the known relationship between peripheral inflammation, brain inflammation and disturbances in cognitive function, mood and behavior in non-cancer contexts (10–16). Given that numerous studies have identified an increase in proinflammatory cytokines in response to chemotherapy, it is not surprising that inflammation would be considered a candidate mechanism (17–19).

If inflammation is causal in chemotherapy-induced cognitive impairment and mood disorders, then prophylactic and sustained treatment with anti-inflammatory drugs would be a potential intervention strategy to prevent these symptoms in cancer patients. We have previously reported that the anti-inflammatory drug aspirin prevents *tumor*-induced cognitive impairment in a mouse model of metastatic breast cancer (11), suggesting its potential to be given to patients prior to chemotherapy to reduce cognitive impairment induced by the cancer. To date, no one has tested the utility for aspirin to combat chemotherapy-induced cognitive impairment. Here we determine if aspirin may also serve to alleviate chemotherapy-induced cognitive impairment. We test the immediate and sustained impact of a commonly employed chemotherapeutic agent, paclitaxel, on memory performance and depression-like behavior in non-tumor bearing mice, and examine the inflammatory profile of these 24 h after the last dose of paclitaxel and 2 weeks later. Paclitaxel is a commonly-used treatment for multiple cancer types including breast, ovarian, lung, bladder, and prostate cancer that has been associated with symptoms of cognitive impairment and mood disorders in cancer patients (20, 21). Paclitaxel has also been shown to alter plasma inflammatory cytokines in patients (22). Consistent with those clinical observations, we show evidence of peripheral inflammation, as indicated by increased plasma cytokines 2 weeks after paclitaxel treatment, but no evidence of elevated cytokines in the brain at any time point. The findings suggest that inflammation may not be responsible for either the immediate or late effects of paclitaxel on cognition, and confirm that aspirin is an unlikely intervention strategy to prevent or treat paclitaxel-induced chemobrain.

## Materials and Methods

### Animals

Experiments used BALB/c male (n = 52) mice aged 13–14 weeks (Monash University, Australia). Mice were individually housed in standard shoebox cages in a temperature and humidity-controlled environment with a 12/12-h modified dark-light cycle (lights on at 1900 h). Food and water were available *ad libitum*. Mice were handled daily for 2 weeks prior to the start of the experiment. Mice were euthanized with CO_2_ either 24 h after cessation of paclitaxel treatment or 2 weeks later to examine biological markers corresponding to early vs. late effects of chemotherapy. Blood was collected by cardiac puncture and tissues were removed after perfusion with sterile PBS and snap frozen in liquid nitrogen. All procedures involving mice were carried out under protocols approved by the Monash Institute of Pharmaceutical Sciences Animal Ethics Committee and in accordance with National Health and Medical Research Council animal ethics guidelines. All experiments were conducted Monash Institute of Pharmaceutical Sciences except for assessment of brain cytokine gene expression, which was conducted at Neuroscience Research Australia.

### Treatments

#### Paclitaxel Treatment

Mice were injected intraperitoneally with 10 mg/kg paclitaxel (Assay Matrix) solubilized in vehicle (Cremorphor EL:ethanol:saline, 1:1:10) or an equivolume of vehicle every second day for 2 weeks.

#### Anti-Inflammatory Treatment

BALB/c mice were treated with either aspirin in drinking water (Aspro, Bayer Australia Ltd) (0.125 µg/ml) or water (control) starting 24 h prior to the first injection of paclitaxel and continuing until the end of the experiment. Water was changed daily and liquid consumption quantified. Mice were pre-determined to drink an average of 4 ml/day of aspirin-treated and non-aspirin-treated water resulting in an estimated dose of 25 mg/kg/day for mice treated with aspirin. This dose produces a similar pharmacodynamic effect on COX-1 inhibition as a dose of 100 mg/day in humans, which is considered a low dose (23).

### Behavioral Assays

Sickness behavior, memory and depression-like behavior were assessed in response to treatment with paclitaxel. To ensure mice received adequate access to aspirin throughout the entire experiment, only sickness and memory was assessed in response to aspirin in drinking water.

#### Sickness

The effect of paclitaxel on sickness and inactivity was assessed by measuring body weight changes, daily burrowing, and locomotor activity. The burrowing test was performed daily at 0900 as described previously (11). A PVC tube filled with 200 ± 1 g of standard mouse chow pellets was placed into the home cage for 30 min. The food pellets remaining in the tube after 30 min were weighed and used to calculate the burrowing activity as a percentage using the formula:

$$\frac{200g-amount\;of\;food\;pellet\;left\;(g)}{200g}\times 100$$

Mice were trained to burrow at least 80% of the pellets (160 g) prior to the start of paclitaxel treatment.

Locomotor activity was assessed by placing mice in a 40 cm × 40 cm × 40 cm testing arena and recorded for 5 min in complete darkness under infrared lighting. Distance travelled was analyzed using Viewer III software (Biobserve GmbH, Bonn, Germany).

#### Cognition and Mood Testing

Episodic memory was assessed using the novel object/novel place recognition test. Novel object/novel place recognition was conducted under low lighting, as previously described (11). The novel object/novel place recognition test was performed in a testing arena (40 cm × 40 cm × 40 cm) 24 h after the termination of paclitaxel treatment and 13 days later. This test comprises of 3 different phases—acquisition, retention, and the test phase. During the acquisition phase, mice were allowed to explore two identical objects for 5 min. Mice were then removed from the arena and placed back into their home cage for 5 min (retention phase). During the test phase, mice were returned to the arena with one of the original now-familiar objects replaced with a novel object (with similar dimensions), placed in a novel location in the arena. BALB/c mice have been shown to perform poorly on the novel object recognition test compared to other strains. BALB/c were chosen to align with previous studies demonstrating cancer-induced memory impairment for which inflammation was shown to be causal and preventable with aspirin (11). Pilot studies indicated that a retention phase of 5 min provides optimal novel object/novel place preference in BALB/c mice while allowing deficits in memory from chemotherapy to be reliably observed. Mouse exploratory behavior, including time spent exploring both old and novel objects and distance travelled, were recorded and analyzed using Viewer III software (Biobserve GmbH, Bonn, Germany). Novel object recognition was calculated by determining the percentage of time spent with the novel object divided by the total time spent exploring all the objects in the arena. Mice were excluded if they spent less than 20 s exploring the familiar objects in the acquisition phase. As the same cohort of mice were used in the test at both time points (24 h and 13 days after paclitaxel), different objects were used between these tests to ensure novelty of the objects to mice.

Working memory was assessed using the Y-Maze spontaneous alternation test and was conducted 24 h after novel object/novel place recognition testing (i.e. 48 h after paclitaxel and 11–13 days thereafter) under dim lighting. Mice were placed in the middle of a three-arm Y-Maze (20.5 cm × 11.5 cm × 32 cm; h × w × l) and were allowed to explore all three arms freely for 5 min. The experiment was recorded by camera and the number and sequence of arm entries were scored manually. Arm entries were only scored when all four limbs of the mice entered the arm. The percentage of correct alternations was calculated using the following formula:

$$\frac{Number\;of\;correct\;alternations\;\left(consecutive\;entries\;into\;three\;different\;arms\right)}{total\;possible\;correct\;alternations\;\left(i.e.,\;total\;arm\;entries-2\right)}\times 100$$

All mice achieved >60% alternation at baseline.

Depression-like behavior was assessed using the sucrose preference and forced swim tests. Sucrose preference was assessed by providing mice with access to two water bottles overnight on a daily basis throughout the experiment. One bottle contained drinking water and the other bottle contained 1.5% (w/v) sucrose in drinking water. The percentage of sucrose solution over the total liquid consumed was calculated. The amount of plain water and sucrose solution consumed was weighed daily. Prior to the start of paclitaxel, mice were trained to achieve 80% or higher of daily sucrose consumption. Behavioral testing for memory, where mice were removed from their home cage, interfered with liquid consumption and sucrose preference testing, making the data unreliable. As such we excluded sucrose preference data during the days of behavioral testing for memory and locomotor activity that required removal from the home cage.

The forced swim test was conducted 1 h after the completion of the novel object/place recognition test. Mice were placed individually in a plastic bucket (20 cm diameter) filled to a depth of 15 cm with water (24 ± 1°C) for 6 min. After the testing period, mice were removed and dried with paper towel before being returned to their home cage. The test was performed in complete darkness and recorded under infrared light. The total swimming distance was tracked and measured using Viewer III (Biobserve GmbH, Bonn, Germany) software. Immobility time was scored manually by an observer blinded to treatment condition.

### Gene Expression Analyses

Total RNA was isolated from frozen tongue, whole brain, and liver tissue using NucleoSpin^®^ Plus (Nucleospin, Germany). Real time reverse transcriptase polymerase chain reaction (qRT-PCR) was used to quantify gene expression in 100 ng of total RNA using Taqman probes (Applied Biosystems, USA) targeting mouse genes of interest: *Tas1r1* Mm00473433_m1, *Tas1r2* Mm00499716_m1, *Tas1r3 Mm00473459_g1*, *Il-1b* Mm01336189_m1, *Tnfa* Mm00443258_m1, *Il-6* Mm00446190_m1, *Ifng* Mm01168134_m1, *Ido1* Mm00492590_m1, *Actb* Mm00607939_s1), and an iScript One-Step RT-PCR kit (Biorad, USA), with 40 PCR amplification cycles of 15 s of strand separation at 95°C, and 30 s of annealing and extension at 60°C. Relative quantitative measurement of target gene levels was performed using the ΔΔCt method, where Ct is the threshold concentration. *Actb* was used as the endogenous housekeeping control gene for livers, brain and tongue.

### Quantification of Tongue Papillae

Fresh tongue tissue was harvested from mice immediately after CO_2_ euthanasia. The dorsal surface of the tongue was immediately stained with India ink to enhance visualization of tongue papillae. Images of tongue tissues were captured using a dissecting microscope with camera attachment. A defined and consistent virtual circular region was drawn around the anterior part of the tongue in each image and the number of papillae within the region was scored by an observer blinded to treatment condition.

### Plasma and Brain Cytokine Protein Analysis

Blood samples were centrifuged at 5000 g for 10min at 4°C. Plasma was isolated and stored at -80°C until assayed. Plasma samples were analyzed undiluted. Frozen whole brains were first pulverized using sterilized steel tissue crushers, which were placed in liquid nitrogen prior to use to ensure tissue temperature did not rise. The powder was thoroughly mixed to ensure equal representation of brain regions throughout the powder. Protein was then extracted from 20 mg of the frozen brain tissue sample by sonicating samples in 200 µl extraction solvent (PBS containing 1xPhosSTOP, Roche). Samples were centrifuged at 15,500 g for 15 min at 4°C and supernatant was collected. Protein content was measured using Bradford assays and standardized to 2 mg/ml A panel of 23 cytokines was measured, using a Bioplex Pro Mouse Cytokine 23-plex Assay kit (Bio-Rad, Gladesville, Australia), according to the manufacturer’s protocol. The plate was read using a Bio-Rad BioPlex200 machine. Median fluorescence data was collected by the instrument and the Bio-Plex Manager software calculated cytokine concentrations as pg/ml based on the standard curve per cytokine using a 5-parameter logistic (5-PL) method.

### Plasma and Brain Paclitaxel Concentration Analysis

Paclitaxel concentration in the brain and plasma of mice (n = 4) was determined by LC-MS using a Waters Micromass Quattro Premier coupled to a Waters Acuity UPLC. Gradient chromatography was achieved with an acetonitrile-water/0.05% formic acid mobile phase and a Supelco Ascentic Express RP C18 (50 x 2.1 mm, 2.7 µm) column. Each whole brain was homogenized in aqueous buffer (3-fold mass/volume ratio), and the resulting homogenates were then protein-precipitated with acetonitrile (3-fold volume/volume ratio). Plasma samples were protein-precipitated with acetonitrile (2-fold volume/volume ratio). Quantitation was performed relative to spiked calibration standards prepared in blank mouse plasma (paclitaxel range: 1–10,000 ng/ml) and brain homogenate (paclitaxel range: 5-10,000 ng/g tissue).

### Statistical Analysis

One-way or two-way repeated measures analyses of variance (ANOVA) (paclitaxel vs vehicle) (aspirin vs placebo) were used to analyze longitudinal data assessing memory, depression-like behavior, and sickness responses and behavior. Single time-point data (cytokines, tongue papillae, and taste receptors) were assessed using unpaired t-tests. Planned comparisons were performed to assess change from the control group, and were determined using Newman-Keuls multiple comparisons tests adjusted for the number of comparisons to control for family-wise error. Association between plasma cytokines at day 26 and memory performance on the novel object/place recognition test at day 22 of the experiment was examined using the Pearson correlation test. Outliers were considered if they were 2 or more standard deviations from the mean. Experiments were conducted in duplicate or triplicate. The data underlying this study are available upon request to the corresponding author.

## Results

### Paclitaxel Causes Sustained Deficits in Novel Object/Novel Place Recognition and Reductions in Sucrose Preference

**Figure 1:**
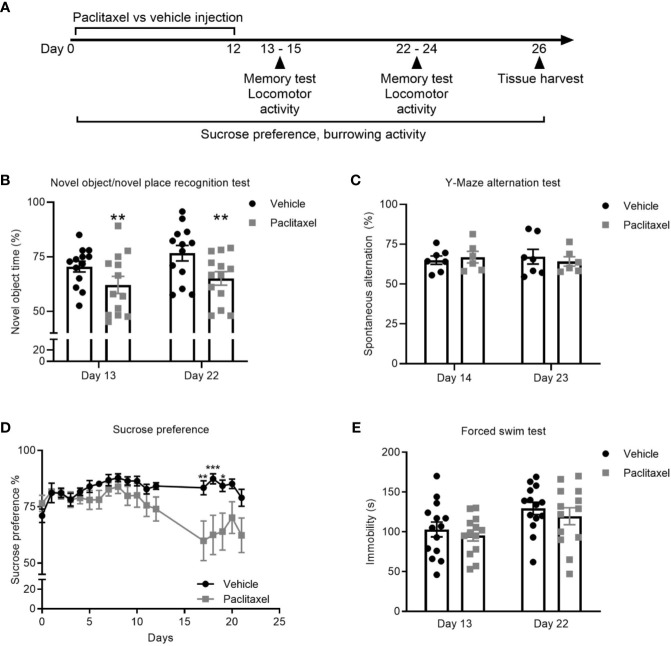
Paclitaxel induces memory impairment and anhedonia. **(A)** Experimental design to evaluate the immediate and late effects of paclitaxel on memory and depression-like behavior for mice treated with paclitaxel vs. vehicle. Mice were treated every second day with 10 mg/kg paclitaxel vs. vehicle for 14 days and memory was assessed at 24–48 h and 10–11 days after the final dose of paclitaxel (days 13–14 and 22–23 of the experiment). **(B–C)** Memory assessed in the test phase of the novel object/novel place recognition test or in the Y-Maze alternation test. **(D)** Depression-like behavior was assessed by sucrose preference throughout and after treatment with paclitaxel. **(E)** Immobility was assessed in the forced swim test at 24 h and 10 days after paclitaxel treatment (days 13 and 22 of the experiment). Data are presented as mean + SE; n = 6–16/group. *p < 0.05; **p < 0.01 from repeated two-way ANOVA (**B**—main effect of paclitaxel) or repeated one-way ANOVA followed by Sidak’s multiple comparison test **(D)**.

To explore the effect of chemotherapy on memory, we treated mice with paclitaxel and used the novel object/novel place recognition and Y-Maze spontaneous alternation tests (**Figures 1A–C**). The novel object/novel place recognition test assesses memory by capitalizing on the innate preference of mice to explore novelty. It requires mice to remember an object they saw, and its position, in a previous period of exploration. Paclitaxel caused immediate and sustained impairment (within 24 h of stopping treatment, i.e. on day 13 of the experiment) on the novel object/novel place recognition test, indicated by reduced time spent exploring the novel object compared to vehicle-treated controls. Cognitive impairment was sustained 10 days after cessation of chemotherapy (day 22 of the experiment; *F*_(1,24)_ = 15.48, *p* = 0.01) (**Figure 1B**). The Y-Maze spontaneous alternation test assesses spatial reference memory and working memory (24). No difference in working and spatial reference memory was observed at either time point based on performance on the Y-Maze spontaneous alternation test (*p* > 0.05) (**Figure 1C**).

**Figure 2:**
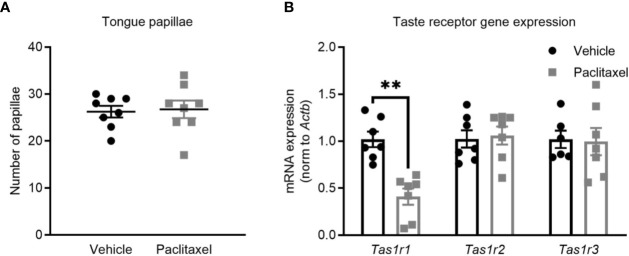
Paclitaxel does not impair sweetened taste perception. **(A)** Tongue papillae number (mean + SE) of mice treated with paclitaxel vs. vehicle. **(B)** Taste receptor mRNA expression of mice treated with paclitaxel vs. vehicle. Data are presented as mean + SE; n = 7–8/group. **p < 0.001 from two-tailed unpaired Student’s t-test.

Depression-like behavior was assessed using the sucrose preference and forced swim tests. Both tests are sensitive and well-validated methods, where depression-like behavior in rodents is indicated by reduced consumption of sucrose solution or increased immobility during the forced swim test (25, 26). Paclitaxel reduced sucrose preference, which reached significance based on multiple comparisons on days 17-19 (*F*_(19,540)_ = 3.17, *p* < 0.0001) (**Figure 1D**) but did not change forced swim test immobility (**Figure 1E**) (*p* > 0.05). Reduced sucrose preference could indicate depression-like behavior, or may be attributable to chemotherapy-induced changes in taste perception, which would not impact behavior in the forced swim test. To investigate this, we quantified the effect of chemotherapy treatment on taste bud density and taste receptor expression. Patients report a loss of taste perception after chemotherapy (27, 28), which has been attributed to loss of taste buds and the receptors that signal flavor perception (29, 30). Perception of sweet flavors are determined by receptors encoded by *Tas1r2* and *Tas1r3* genes, whereas umami flavors involve *Tas1r1* and *Tas1r3* genes. Paclitaxel had no effect on the quantity of tongue papillae (**Figure 2A**), but reduced expression of *Tas1r1* mRNA [*t*_(12)_ = 5.12, *p* < 0.01] without affecting either *Tas1r2* or *Tas1r3* gene expression, indicating a possible impairment in umami flavor perception but not for perception of sweet flavors such as sucrose solution (**Figure 2B**). Considered together, these data suggest that the observed reduction in sucrose preference reflects paclitaxel-induced anhedonia and not an inability to recognize the sweet sucrose solution.

### Paclitaxel-Induced Memory Impairment and Anhedonia Are Independent of Paclitaxel-Induced Sickness

**Figure 3:**
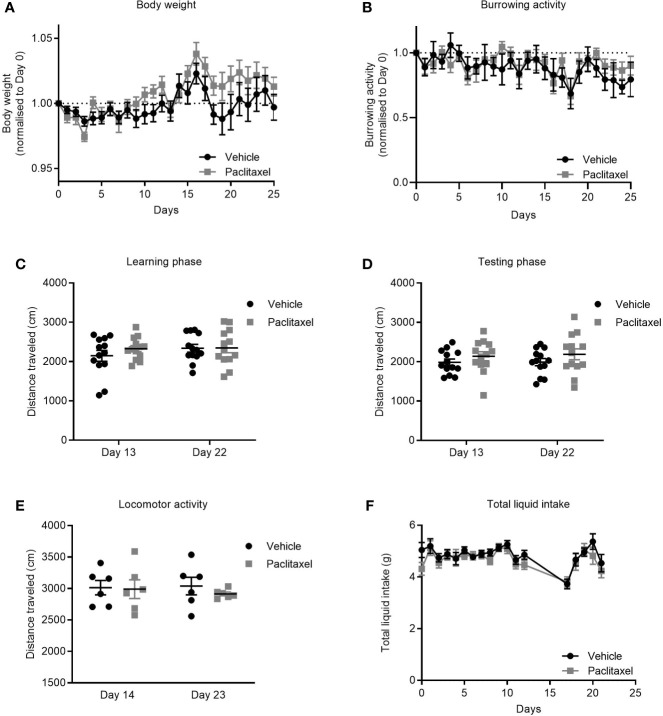
Paclitaxel does not induce sickness or inactivity. **(A)** Body weight change from baseline for mice treated with paclitaxel vs vehicle throughout the experiment. **(B)** Burrowing activity change from baseline for mice treated with paclitaxel vs. vehicle throughout the experiment. **(C–D)** Distance travelled in the novel object/novel place recognition test during learning and testing phases for mice treated with paclitaxel vs vehicle. **(E)** Spontaneous locomotor activity for mice treated with paclitaxel vs. vehicle. **(F)** Total liquid intake (g) for mice treated with paclitaxel vs. vehicle throughout the experiment. Data are presented as mean + SE; n = 6–16 mice/group.

Cancer patients receiving chemotherapy typically report symptoms of sickness such as nausea, weight loss, and fatigue that are attributed to both the cancer and to chemotherapy treatment (31, 32). To investigate if paclitaxel-induced memory impairment and anhedonia (**Figures 1B, D**) were due to treatment-induced inactivity and/or sickness, we assessed the effect of paclitaxel on body weight, liquid consumption, and burrowing activity. Paclitaxel did not affect body weight (**Figure 3A**) or burrowing activity (**Figure 3B**), and did not alter distance travelled in either the learning or testing phase in the novel object/novel place recognition (**Figures 3C, D**) or in the locomotor activity test (**Figure 3E**). These findings indicate that poor performance on the behavioral tasks was not due to inactivity or loss of motivation. Furthermore, no difference in liquid consumption was observed between paclitaxel and vehicle-treated mice in sucrose preference test (**Figure 3F**) (*p* > 0.05). Together, these data suggest that the observed memory impairment and anhedonia (**Figures 1B, D**) are unlikely due to paclitaxel-induced sickness behaviors.

### Paclitaxel Induces Delayed Peripheral Inflammation

**Figure 4:**
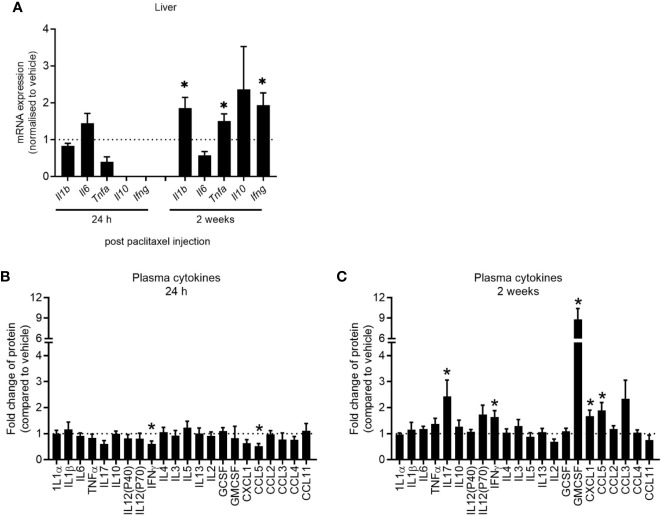
Paclitaxel induces delayed peripheral inflammation. **(A)** Hepatic cytokine mRNA expression of mice 24 h and 2 weeks after paclitaxel treatment (mean + SE). Fold change relative to vehicle-treated mice. (**B–C)** Plasma cytokine concentrations 24 h and 2 weeks after paclitaxel treatment (mean + SE). Fold change relative to vehicle-treated mice. The dotted line at 1 represents vehicle-treated control levels. Data are presented as mean + SE; n = 4–12/group. *p < 0.05 from two-tailed unpaired Student’s t-test.

Clinical studies have reported elevations in pro-inflammatory cytokines in patients and survivors treated with chemotherapy (32–34). We first determined whether our model of paclitaxel-induced memory impairment recapitulated cytokine changes found in those clinical studies by quantifying cytokine gene expression and protein levels 24 h and 2 weeks after stopping chemotherapy treatment. Hepatic cytokines were assayed as a source of peripheral inflammatory cytokines and did not change 24 h after the last dose of paclitaxel as measured by qRT-PCR (p > 0.05) (**Figure 4A**). Plasma protein levels confirmed no evidence of inflammation in paclitaxel-treated mice at this time-point, although IFNγ and CCL5 were significantly reduced (p < 0.05) (**Figure 4B**). However, paclitaxel significantly elevated expression of hepatic *Il1b*, *Tnfa* and *Ifng* 2 weeks after treatment (all p < 0.05) (**Figure 4A**). Inflammation at 2 weeks after cessation of paclitaxel treatment was confirmed with increases in IL17, IFNγ, GMCSF, CXCL1, and CCL5 plasma protein (all p < 0.05) (**Figure 4C**). Correlation analyses confirmed that peripheral cytokines were not associated with memory performance (**Supplementary Table 1**). These findings show that immediate deficits in memory and mood (within 24 h of stopping paclitaxel) were independent of peripheral inflammation. In contrast, sustained memory impairment and anhedonia (2 weeks after treatment cessation) was accompanied by peripheral inflammation.

### Paclitaxel Does Not Induce Neuroinflammation

**Figure 5:**
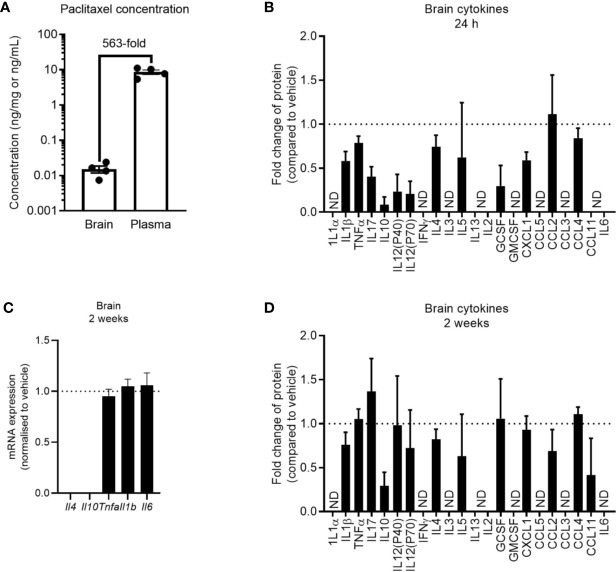
Paclitaxel does not induce brain inflammation. **(A)** Concentration of paclitaxel in brain and plasma 24 h after the final dose of paclitaxel. **(B)** Brain cytokine protein concentrations 24 h after paclitaxel treatment. Fold change relative to vehicle-treated mice. **(C)** Brain cytokine mRNA expression of mice 2 weeks after paclitaxel treatment. Fold change relative to vehicle-treated mice. **(D)** Brain cytokine protein concentrations 2 weeks after paclitaxel treatment. Fold change relative to vehicle-treated mice. Dotted lines at 1 represent vehicle-treated control levels. Data are presented as mean + SE; n = 4–12/group. N.D. = not detectable.

Despite seeing no evidence of peripheral inflammation at 24 h after paclitaxel treatment, it was possible that paclitaxel may be entering the brain and inducing compartmentalized neuroinflammation at this earlier time. While significantly lower in concentration than in the plasma, we still confirmed that paclitaxel crossed the blood brain barrier and was present in the brain parenchyma (**Figure 5A**). We then assessed brain cytokine levels at 24 h and 2 weeks after stopping chemotherapy. Paclitaxel did not increase cytokine or chemokine concentrations at 24 h, but rather decreased several cytokines and chemokines compared to vehicle controls (**Figure 5B**). Paclitaxel treatment had no effect on cytokine gene expression at 2 weeks (p > 0.05) (**Figure 5C**), which was confirmed by protein analysis of a wider array of cytokines and chemokines (**Figure 5D**). These data indicate that the paclitaxel-induced peripheral inflammatory signature may not be reflected in the brain, and that there is no association between neuroinflammation and paclitaxel-induced memory impairment.

### Inflammation Is Not Causal in Paclitaxel-Induced Cognitive Impairment

**Figure 6:**
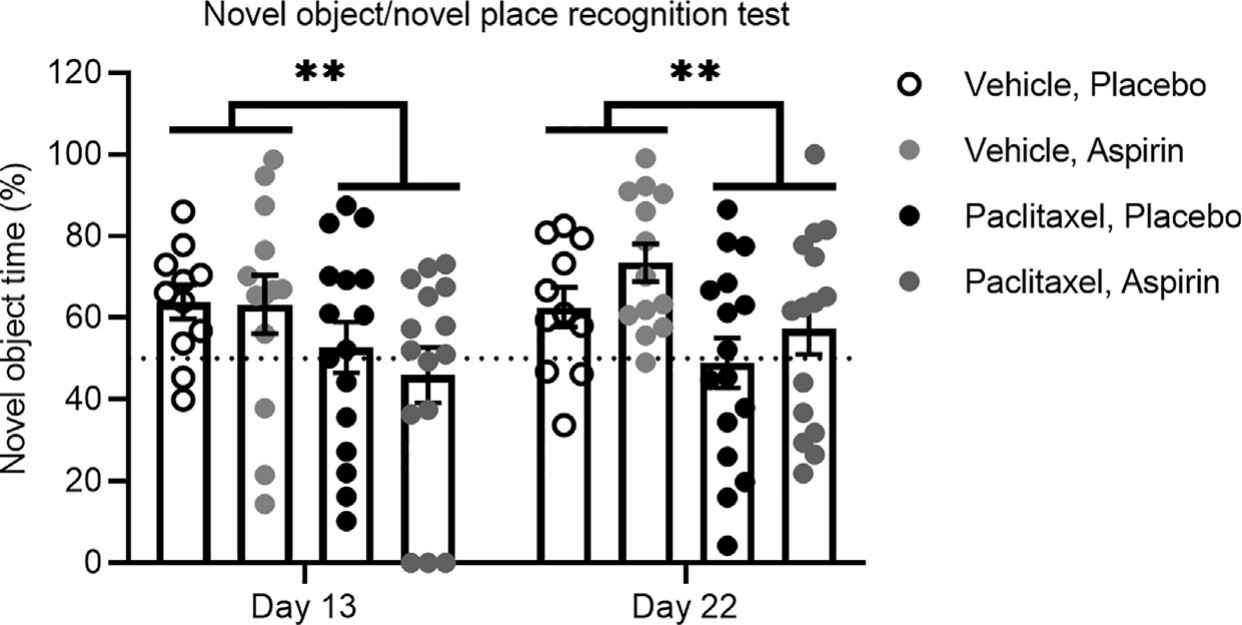
Aspirin does not improve paclitaxel-induced memory impairment. Memory was assessed at 24 h and 10 days after the final dose of paclitaxel or vehicle (days 13 and 23 of the experiment) in the test phase of the novel object/novel place recognition test in aspirin vs. placebo treated mice (n > 11/group). Data are presented as mean + SE; n = 11–16/group. **p < 0.01 from repeated two-way ANOVA (main effect of paclitaxel).

To experimentally confirm that inflammation does not play a role in paclitaxel-induced memory impairment, we treated mice orally with the anti-inflammatory drug aspirin in drinking water at a dose we previously showed to be effective in preventing tumor-induced memory impairment using the novel object/novel place recognition test (11). Mice commenced oral aspirin 24 h prior to the first injection of paclitaxel and were maintained on aspirin until the end of the experiment. To avoid confounds caused by aspirin delivered in drinking water, we did not conduct sucrose preference testing in these mice. Consistent with our previous findings, paclitaxel induced impairment on the novel object/novel place recognition test 24 h after cessation of paclitaxel (day 13 of the experiment), and this was sustained 10 days later (day 23 of the experiment) [*F*_(1,52)_ = 9.07, *p* < 0.01]. Aspirin did not affect memory performance on this task at any time point (**Figure 6**). These findings support the contention that inflammation is not responsible for either the immediate or sustained effects of paclitaxel on memory or affective behavior in this model.

## Discussion

The role of inflammation in chemotherapy-induced cognitive impairment and affective symptoms is controversial. While multiple clinical studies have reported associations between peripheral inflammatory markers and reduced mood and cognitive impairment in cancer patients receiving chemotherapy, this correlation could plausibly be explained by effects of the chemotherapy or the cancer itself (11). By using animal models, the current study eliminated the potential confound of cancer-induced inflammation on memory and mood-related behaviors and revealed the role of paclitaxel chemotherapy on behavior and inflammation in the body and the brain. In doing so, this study replicated clinical studies that demonstrate peripheral inflammatory markers occur alongside mood and cognition changes in patients that receive chemotherapy (32–34), suggesting that paclitaxel alone may contribute to the increase of these inflammatory markers. However, the findings of this study support the contention that pro-inflammatory cytokines are associated with, but not responsible for, chemobrain.

Not all cognitive and depression-like domains were impaired by paclitaxel. The novel object/novel place recognition test involves aspects of both episodic and spatial memory, whereas the spontaneous Y-Maze alternation test measures working and spatial memory. Paclitaxel-induced impairment in the novel object/novel place test suggests that hippocampal-dependent episodic memory is vulnerable to paclitaxel whereas heavily-utilized working memory and spatial memory may be more resilient to the impact of paclitaxel. This contention is supported by studies showing that the hippocampus is sensitive to impairment in neurogenesis by paclitaxel (35, 36). Similarly, while paclitaxel induced anhedonia, it did not cause other depression-like phenotypes such as learned helplessness in the forced swim test, suggesting certain domains of depression may be more susceptible to the impact of chemotherapy than others. Future studies should explore the possibility that the changes in sucrose preference reflect anhedonia specifically and not paclitaxel-induced changes to the interoceptive state of the mice, which are partially refuted by the examination of tongue papillae and taste receptor gene expression in this study. These findings confirm clinical studies that report differential effects of chemotherapy on numerous cognitive processes and across time (37, 38). Future evaluation of specific brain regions, as well as affective and cognitive processes, are required to better understand the impact of chemotherapy on cognition.

Clinical studies have reported elevations in pro-inflammatory cytokines in the blood of patients and survivors treated with chemotherapy (32–34). This has led to the assumption that neuroinflammation is induced by chemotherapy-induced peripheral inflammation, which propagates inflammation to the brain. Consistent with clinical findings, paclitaxel caused immediate and long-lasting impairment in memory and anhedonia in mice, but peripheral inflammation only became apparent 2 weeks after paclitaxel treatment. Regardless, peripheral cytokines measured 2 weeks after paclitaxel did not propagate inflammation to the brain (**Figure 5D**), which disputes the assumption made in patients that chemotherapy-induced inflammation in the body is always reflected in the brain. There was also no evidence of inflammation in the brain at 24 h after paclitaxel treatment despite evidence of memory impairment and anhedonia, and paclitaxel having crossed the blood-brain barrier. These findings are discordant with the contention that transit of paclitaxel into the brain induces compartmentalized neuroinflammation, leading to memory loss and affective symptoms in cancer patients who receive chemotherapy.

The absence of inflammation after chemotherapy reported here is supported by other preclinical studies (39, 40). Nonetheless, some studies have shown that chemotherapy increases inflammatory cytokines in blood and brain tissue (18, 19, 41–43). As each of these animal studies used different chemotherapeutic agents, doses, or treatment regimens, it is possible the role of inflammation on chemobrain may be specific to chemotherapy type or magnitude of exposure.

The fact that increases in cytokines were not observed in plasma until 2 weeks after paclitaxel treatment despite immediate effects on memory and anhedonia is supported by clinical data. In breast cancer patients no significant changes in plasma inflammatory cytokines were observed until 3 weeks after cessation of the entire chemotherapy regimen, despite impairment in perceived cognitive function being reported during chemotherapy treatment (8). Together these findings suggest a temporal dissociation between chemotherapy-induced cytokine production and symptoms of chemobrain, and supports the contention that alternative mechanisms are responsible for the induction of at least some symptoms of chemobrain.

While the results do not support a role for cytokines in chemobrain, it is possible that other inflammatory mediators such as prostaglandins contribute to chemobrain. Paclitaxel has been shown to affect macrophages to induce the expression of cyclooxygenase-2—the enzyme that converts arachidonic acids to prostaglandins (44)—and increase the production of prostaglandin E_2_ (45). As prostaglandins have been implicated in the progression of several neurodegenerative diseases (46), it is possible that paclitaxel may similarly increase production of prostaglandins in mice, contributing to memory impairment. However, administration of aspirin, a non-selective anti-inflammatory drug that blocks cyclooxygenase-2, had no effect on paclitaxel-induced memory impairment in the current study, supporting the contention that inflammation may not contribute to paclitaxel-induced memory impairment. These findings also indicate that while aspirin may have therapeutic utility for the prevention of tumor-induced cognitive impairment (11), aspirin is unlikely to show benefit in targeting paclitaxel-induced chemobrain. It will be important to determine the potential use of aspirin to target chemobrain symptoms induced by other chemotherapeutic agents and dosing schedules that have been shown to increase inflammatory cytokines (19, 41–43).

Study limitations may have influenced the capacity to observe noticeable changes in cytokine levels in paclitaxel-treated mice. Firstly, we assessed cytokines in whole brains, which may mask the effects of chemotherapy on cytokines in specific brain regions (41). Secondly, it is possible that the method of euthanasia influenced cytokine levels, which has been previously reported (47). However, all mice were euthanized using the same method.

The current findings offer important insights into correlative evidence that cognitive impairment and mood-related symptoms are associated with elevated inflammatory markers in clinical (6, 34) and preclinical studies of cancer treatment (19, 48). This study confirms recent findings that paclitaxel is able to cross the blood brain barrier in the absence of cancer (35), raising the possibility of direct cytotoxic effects of paclitaxel on cells of the brain. Recent studies support this assertion with evidence that chemotherapy drugs induce apoptosis in neurons, reduce neurogenesis and synaptic integrity, and cause mitochondrial dysfunction (19, 40, 42, 48, 49). Additionally, the downregulation of vesicular zinc in the hippocampus has also been shown to mediate the effect of paclitaxel on hippocampal neurogenesis (36). Moreover, the presence of cancer could enhance “leakiness” of the blood-brain barrier (50), thereby potentiating the penetration of paclitaxel to the brain and the magnitude of neurotoxicity. However, as chemotherapy could reduce cancer burden, chemotherapy may equally reduce the effect of cancer-induced cognitive impairment (11). Emerging studies examining the effects of both chemotherapy and cancer on cognitive impairment will be able to provide more insights into the complex interaction between chemotherapy, cancer and the brain (40).

## Conclusion

Our findings question the role of inflammation in paclitaxel-induced chemobrain. The findings show aspirin is unlikely to be effective in preventing or treating chemotherapy-induced cognitive impairment, in contrast to recent findings that aspirin may be effectively repurposed to combat *cancer*-induced cognitive impairment (11). This distinction is critical to inform clinical trial design for evaluation of anti-inflammatory drugs to treat cognitive impairment, before, during, and after cancer treatment.

## Data Availability Statement

The raw data supporting the conclusions of this article will be made available by the authors, without undue reservation.

## Ethics Statement

The animal study was reviewed and approved by Monash Institute of Pharmaceutical Sciences Animal Ethics Committee.

## Author Contributions

AC contributed to conception, design, data acquisition, analysis, interpretation, and manuscript preparation. NC contributed to data acquisition, analysis, interpretation, and manuscript preparation. AL contributed to data acquisition, analysis, and manuscript preparation. AZ was involved in data acquisition and analysis of plasma cytokines. DS was involved in data acquisition and analysis of brain and plasma paclitaxel concentrations, and manuscript preparation. ES was involved in design, data analysis, interpretation, and manuscript preparation. AW was involved in all aspects of this study including conception, design, data acquisition, analysis, and interpretation and preparation of the manuscript. All authors contributed to the article and approved the submitted version.

## Funding

This work was supported by the National Breast Cancer Foundation, Australia [PF-15-014] and the National Health and Medical Research Council [1147498], and the Schizophrenia Research Institute and Neuroscience Research Australia (NeuRA).

## Conflict of Interest

The authors declare that the research was conducted in the absence of any commercial or financial relationships that could be construed as a potential conflict of interest.

## Supplementary Materials

Supplementary Table 1 Correlation between plasma cytokines and memory performance on the novel object/novel place recognition test ~ 2 weeks after paclitaxel.
