# Supplementary material for: The Anti-Inflammatory Drug Aspirin Does Not Protect Against Chemotherapy-Induced Memory Impairment by Paclitaxel in Mice
Source: Front Oncol. 2020 Dec 14;10:564965. doi: 10.3389/fonc.2020.564965 (PMC7768078; doi:10.3389/fonc.2020.564965)
Supplement: Supplementary Table 1 — Correlation between plasma cytokines and memory performance on the novel object/novel place recognition test ~ 2 weeks after paclitaxel. [file DataSheet_1.pdf]

Supplementary Table 1. Correlations between plasma cytokines and memory performance on the novel object/novel place recognition test ~ 2 weeks after paclitaxel.

**Cytokine**	**R**	**R^2**	***P* values**
IL-1a	-0.2142	0.0459	0.4821
IL-1b	-0.1101	0.0121	0.7202
IL-2	-0.0801	0.0064	0.7948
IL-3	-0.1347	0.0181	0.6610
IL-4	-0.1232	0.0152	0.6883
IL-5	-0.2922	0.0854	0.3326
IL-6	-0.0306	0.0009	0.9209
IL-9	0.0569	0.0032	0.8534
IL-10	-0.2176	0.0473	0.4751
IL-12(p40)	0.1067	0.0114	0.7285
IL-12(p70)	-0.1492	0.0223	0.6267
IL-13	-0.2672	0.0714	0.3775
IL-17	-0.0962	0.0092	0.7546
Eotaxin	0.0684	0.0047	0.8242
G-CSF	0.2488	0.0619	0.4123
GM-CSF	-0.2401	0.0576	0.4295
IFN-g	-0.0337	0.0011	0.9130
KC	-0.2641	0.0697	0.3833
MCP-1	-0.1761	0.0310	0.5649
MIP-1a	-0.0114	0.0001	0.9705
MIP-1b	0.0492	0.0024	0.8731
RANTES	-0.0224	0.0005	0.9421
TNF-a	-0.1140	0.0130	0.7109
